# Lymphocyte subset expression and serum concentrations of PD-1/PD-L1 in sepsis - pilot study

**DOI:** 10.1186/s13054-018-2020-2

**Published:** 2018-04-17

**Authors:** Julie K. Wilson, Yuan Zhao, Mervyn Singer, Jo Spencer, Manu Shankar-Hari

**Affiliations:** 1 Department of Intensive Care Medicine, Guy’s and St Thomas’ Hospitals NHS Foundation Trust, London, SE1 7EH UK; 2 Department of Immunobiology, School of Immunology & Microbial Sciences, King’s College London, London, UK; 3 Bloomsbury Institute of Intensive Care Medicine, Division of Medicine, University College London, London, UK

**Keywords:** Sepsis, Septic shock, Programmed death-1, Enrichment, Outcomes

## Abstract

**Background:**

Sepsis remains a major cause of mortality in critical care, for which specific treatments are lacking. The dysregulated response to infection seen in sepsis includes features of lymphocyte dysfunction and exhaustion, suggesting that immune-stimulatory therapy may improve outcomes in certain patient groups. Monoclonal antibodies targeting checkpoint molecules, such as programmed-death 1 protein (PD-1) and its ligand PD-L1, have shown success in stimulating the immune response in patients with cancer, and are being considered for future sepsis trials. The aims of this pilot study were to compare lymphocyte subset expression of PD-1 and its ligands between patients with sepsis and controls; to characterize serum levels of PD-1 and PD-L1 in patients with sepsis and controls, and determine if serum concentrations correlated with cell surface expression.

**Methods:**

Expression levels of PD-1, PD-L1 and PD-L2 on four lymphocyte subsets (CD27 + CD19+ B cells, CD27-CD19+ B cells, CD27 + CD4+ T cells and CD27-CD4+ T cells) were compared between 22 patients with sepsis (including 11 survivors and 11 non-survivors) and 11 healthy controls using flow cytometry. Levels of soluble PD-1 and PD-L1 were also compared using commercially available ELISA kits.

**Results:**

Expression of PD-1 and PD-L1 was higher on all lymphocyte subsets in patients with sepsis compared to controls (*p* < 0.05). PD-L2 expression on CD27+ B cells was also higher in patients with sepsis (*p* = 0.0317). There was differential expression of PD-1 by CD27 status, with expression being higher in the B and T cell subsets associated with memory status (CD27+ and CD27-, respectively; *p* < 0.001). Higher PD-1 and PD-L1 expression was not associated with mortality or with a higher risk of nosocomial infection. There were no differences in levels of soluble PD-1 or PD-L1 between patients with sepsis and controls.

**Conclusions:**

Higher expression of PD-1 by memory subpopulations of B cells and CD4+ T cells, with normal soluble PD-1 and PD-L1 in patients with sepsis, are novel findings. This information may be useful to enrich sepsis populations for trials of PD-1/PD-L1 blockade.

**Electronic supplementary material:**

The online version of this article (10.1186/s13054-018-2020-2) contains supplementary material, which is available to authorized users.

## Background

Sepsis is a dysregulated host response to infection [1], with concomitant immune activation and suppression. Sepsis-related immunosuppression contributes to poor outcomes by increasing the risk of nosocomial infection and death [2–4]. A common feature of sepsis-related immunosuppression is impaired lymphocyte function, with increased expression of inhibitory checkpoint molecules, such as programmed-death 1 protein (PD-1) [2]. PD-1 serves to limit excessive immune responses by negatively regulating lymphocyte activation and function, and promoting immune cell apoptosis. It has two known ligands: programmed death ligand-1 (PD-L1), which is widely expressed by a variety of immune and non-immune cell types; and programmed death ligand-2 (PD-L2), which is expressed by antigen-presenting cells [2]. Increased expression of PD-1 and PD-L1 by T cells, monocytes and neutrophils has been demonstrated in sepsis, while upregulation of the PD-1 pathway is associated with higher mortality [2, 5–8]. As this dysfunction is potentially reversible with anti-PD-1 or anti-PD-L1 monoclonal antibody treatment [2, 5–8], manipulating the PD-1 pathway represents a potential target for sepsis trials.

Against this background, we hypothesized that lymphocyte surface PD-1, PD-L1 and PD-L2 expression by B and T cell subsets will vary by CD27 expression status. CD27 is a marker of lymphocyte activation; CD27 positive (CD27+) B cells correspond to memory B cells [9], while CD27 negative (CD27-) T cells represent a high antigen-recall subset of memory T cells [10]. Another rationale for assessing CD27-based memory lymphocyte subsets is the selective depletion of memory B cells in sepsis [8]; it is not known whether PD-1 expression varies by lymphocyte memory status. We therefore measured PD-1, PD-L1 and PD-L2 surface expression on CD27+ and CD27- subsets of CD4+ T and B lymphocytes using flow cytometry in adult patients with sepsis on the intensive care unit (ICU). We compared expression between patients with sepsis and healthy controls, and between subgroups of patients with sepsis by nosocomial infection and survival status. PD-1 and PD-L1 also exist in a soluble form in serum; however, the relevance of these soluble forms to sepsis pathogenesis is unclear. We hypothesized that cell-surface PD-1, PD-L1 and PD-L2 expression would correlate with serum concentrations, and so we measured corresponding serum PD-1 and PD-L1 levels in the same samples.

## Methods

### Conceptual approach

Immune responses in sepsis differ between patients [3, 4]. The ability to identify who would – or would not – benefit from therapy based on specific biological mechanisms will offer a crucial step forward in patient management, especially when that mechanism is dominant, linked to an outcome of interest, and present at the time of assessment of trial eligibility [11]. These principles informed our study design. Our conceptual approach was that sepsis trial eligibility criteria are often assessed on the day of ICU admission and that patients with increased expression of PD-1 and PD-L1 have a greater risk of nosocomial infections and/or death, as this would be a dominant mechanism contributing to these outcomes.

### Study design and setting

This was an analysis of a subpopulation of patients enrolled into a previous prospective observational cohort study performed in a general medical-surgical tertiary ICU (HRA Research Ethics Committee approval reference: 12/LO/0326). Details of the study design have been published previously [8, 12]. From this cohort we randomly selected 22 adult patients with sepsis, with an ICU length of stay ≥48 h, and included equal numbers of survivors and non-survivors [8, 12]. As our original study was designed prior to the Sepsis-3 definitions, sepsis was identified using the previous definition requiring proven or suspected infection, two or more systemic inflammatory response system (SIRS) criteria, and at least one organ system dysfunction (cardiovascular, respiratory, renal, haematologic, hepatic, neurologic or metabolic) [13]. We highlighted in a recent cohort study that the prevalence of SIRS-negative sepsis in ICU patients in England was approximately 3% [14], with a 92% overlap in sepsis cases identified by Sepsis-2 and Sepsis-3 [15]. We excluded patients less than 18 years old, and those with known immune dysfunction, including those with congenital hypogammaglobulinaemia, known protein-losing enteropathies, nephrotic syndrome and neoplastic or proliferative haematologic diseases; those having received intravenous immunoglobulin therapy in the last 3 months; those receiving high-dose corticosteroid therapy; those with ongoing blood loss (defined by blood transfusion requirement > 2 units in a 24 h period); those with retroviral disease; and those with immune dysfunction as defined by Acute Physiology and Chronic Health Evaluation (APACHE) II score comorbidities [16].

### Blood sampling, flow cytometry, ELISA and healthy controls

Peripheral blood mononuclear cells were isolated by density centrifugation from blood samples collected within 12 h of ICU admission and stored in liquid nitrogen. Serum samples from the same patients were stored at − 80 °C. Anti-human fluorochromes were used to identify lymphocyte subsets: anti-CD19 (PerCP Cy5.5; HB19); anti-CD3 (APC-H7; SK7) (both BD Biosciences, Wokingham, Berkshire, UK); anti-CD4 (Pacific Blue; SK3); anti-CD27 (FITC; 0323); anti-PD-1 (APC; EH12.2H7); anti-PD-L1 (PeCy7; 29E.2A3); and anti-PD-L2 (PE; 24F.101C12) (all Biolegend, London, UK). Amcyan (L34957; Invitrogen, Carlsbad, CA, USA) was used to identify live cells. All flow cytometry experiments were carried out by the same investigator. Flow cytometer set up, calibration and compensation were carried out prior to each experiment using BD CompBeads (BD Biosciences). Reagents remained the same during the course of the study. Gating to identify cell subsets was achieved using isotype controls and fluorescence minus-one (FMO) controls (Additional file 1: Figure S1). FlowJo software (https://www.flowjo.com) was used for analysis of flow cytometry data. Percentage positivity for PD-1, PD-L1 and PD-L2 was defined as the percentage of cells above the gate set using the above controls, with the proportion of positive cells and the corresponding geometric mean fluorescence intensity (MFI) used as indicators of expression. Serum levels of PD-1 and PD-L1 were quantified in duplicate using commercial ELISA kits (Proteintech, Manchester, UK), according to the manufacturer’s instructions. Detection ranges for PD-1 and PD-L1 were 125–8000 pg/ml and 0.156–10 ng/ml, respectively. All flow cytometry and ELISA experiments were based on anonymised healthy controls who gave consent prior to sampling as per the King’s College London Infectious Diseases Biobank protocol [8].

### Statistics

Mann-Whitney U tests were used to compare PD-1, PD-L1 and PD-L2 expression on B and T cells between septic patients and controls, and between subgroups of septic patients based on mortality and nosocomial infection status, with nosocomial infection defined as a new antibiotic start for suspected new infection, after an antibiotic-free period ≥24 h. Within patients with sepsis, PD-1, PD-L1 and PD-L2 expression on B and T cells was compared by CD27 status using the paired Wilcoxon test. All statistical analyses were performed with GraphPad Prism software (GraphPad Software, La Jolla, CA, USA). Significance levels were set as *p* values < 0.05.

## Results

### Study cohort

The median (IQR) age of the septic patients was 68.5 (54.8 – 84.3) years, with 73% male. The respiratory tract was the most common infection site (73%), followed by intra-abdominal (18%) and wound/soft tissue (9%). The mean (SD) total white cell and lymphocyte counts were 15.9 (7.8) × 10^9^ cells/l and 1.1 (0.7) × 10^9^ cells/l, respectively. The median (IQR) ICU length of stay was 10.5 (7–20) days. Nosocomial infection occurred in 11 patients and was more common in patients with an ICU length of stay ≥ 4 days (61% vs 0%). Patient characteristics are summarized in Additional file 2: Table S1 and Additional file 3: Table S2.

### Memory B cells in patients with sepsis had more PD-1, PD-L1 and PD-L2 positive cells with higher expression

**Fig. 1:**
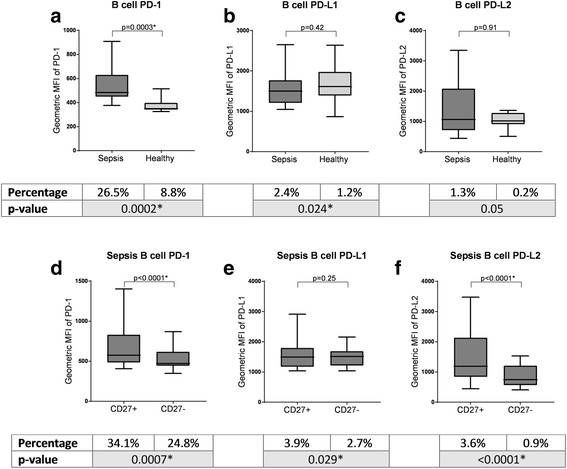
Programmed death protein 1/programmed death ligand 1 (PD-1/PD-L1) expression on B cells. **a**-**c** Comparison of the mean fluorescence intensity (MFI) of PD-1, PD-L1 and PD-L2 on B cells in patients with sepsis compared with healthy controls. **d**-**f** Comparison of the MFI of PD-1, PD-L1 and PD-L2 between CD27+ B cells and CD27- B cells within patients with sepsis. The boxes below show the corresponding proportion of positive cells for each comparison, with corresponding *p* values. *Significant *p* values

The proportion of B cells positive for PD-1 and the corresponding MFI were significantly greater in patients with sepsis than in controls (26.5% vs 8.8%, *p* = 0.0002; 483 vs 348, *p* = 0.0003) (Additional file 4: Table S3; Fig. 1): this was true of both CD27+ and CD27- subsets (Additional file 5: Table S4; Additional file 6: Figure S2; Additional file 7: Figure S3). The proportion of PD-L1 positive B cells was also greater in patients with sepsis compared to healthy controls (2.4% vs 1.2%, *p* = 0.0244). The percentage positivity of PD-L2 was higher in sepsis than controls in the CD27+ subset only (3.59% vs 0.41%, *p* = 0.0317) (Additional file 7: Figure S3). Within B cells in septic patients, the CD27+ subset had significantly higher PD-1 and PD-L2 MFI (574.5 vs 471.5, *p* < 0.0001; 1189 vs 744, *p* < 0.0001) and percentage positivity (34.05% vs 24.80%, *p* < 0.0007; 3.59% vs 0.86%, *p* < 0.0001) (Fig. 1).

### Memory CD4+ T cells in patients with sepsis had more PD-1, PD-L1 and PD-L2 positive cells with higher expression

**Fig. 2:**
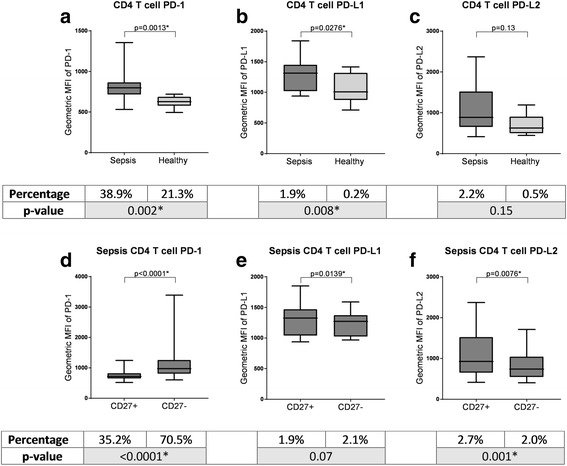
Programmed death protein 1/programmed death ligand 1 (PD-1/PD-L1) expression on CD4+ T cells. **a**-**c** Comparison of the mean fluorescence intensity (MFI) of PD-1, PD-L1 and PD-L2 on T cells in patients with sepsis compared with healthy controls. **d**-**f** Comparison of the MFI of PD-1, PD-L1 and PD-L2 between CD27+ T cells and CD27- T cells within patients with sepsis. The boxes below show the corresponding proportion of positive cells for each comparison, with corresponding *p* values. *Significant *p* values

The proportions of PD-1 and PD-L1 positive T cells were significantly greater in sepsis than in controls (38.90% vs 21.25%, *p* = 0.0023; 1.9% vs 0.2%, *p* = 0.0083), as were the corresponding MFI values (780 vs 627, *p* = 0.0013; 1314 vs 1007, *p* = 0.0276) (Fig. 2): this was true of CD27+ and CD27- subsets (*p* < 0.05 for all comparisons) (Additional file 5: Table S4; Additional file 8: Table S5; Additional file 9: Figure S4; Additional file 10: Figure S5). There were no significant differences in CD4+ T cell PD-L2 expression between patients with sepsis and healthy controls. In patients with sepsis, expression of PD-1 was higher on CD27- T cells than CD27+ T cells, by both percentage positivity and MFI (70.45% vs 35.15%, *p* < 0.0001; 972 vs 716, *p* < 0.0001) (Fig. 2).

### Admission-day PD-1, PD-L1 and PD-L2 expression did not differ by nosocomial infection and hospital mortality

There were no differences in PD-1, PD-L1 or PD-L2 expression between patients who subsequently developed a nosocomial infection and those who did not (Additional file 11: Figure S6). When patients with an ICU length of stay ≤ 7 days were excluded, PD-L1 expression on lymphocytes was significantly higher in those who subsequently developed an infection (*p* = 0.0068) (Additional file 12: Figure S7); however, this was not statistically significant for any B or T cell subset (Additional file 13: Figure S8). There were no differences between survivors and non-survivors in PD-1, PD-L1 or PD-L2 expression for any lymphocyte subset (Additional file 14: Figure S9).

### Soluble PD-1 and PD-L1 expression did not correlate with cell surface expression

There were no differences in serum soluble PD-1 (sPD-1) and sPD-L1 concentrations between patients with sepsis and healthy controls (Additional file 15: Figure S10). There was no correlation between serum sPD-1 and sPD-L1 concentrations in patients with sepsis and lymphocyte surface expression (Additional file 16: Figure S11).

## Discussion

The novel findings from this pilot study include the first report of higher B cell expression of PD-1, PD-L1 and PD-L2 in sepsis, and differential expression of PD-1 by CD27 status in both B and CD4+ T cells. We also report results that are consistent with the published literature such as higher PD-1 and PD-L1 expression in CD4+ T cells in sepsis compared with controls [7, 17], which gives external validity to our report. The overexpression of these checkpoint inhibitors in most patients with sepsis is consistent with the published literature suggesting this is a feature of sepsis-related immunosuppression.

PD-L2 has been less well-studied in sepsis than PD-L1, as PD-L1 is the more important binding partner for PD-1. The contribution of PD-L2 to the pathophysiology of sepsis remains unknown although increased PD-L2 expression by monocytes was reported in one observational study of patients with septic shock [7]. A key finding of our present study in critically ill adult patients with sepsis was higher expression of PD-1/PD-L in the lymphocyte subsets associated with memory status, i.e. CD27+ B cells and CD27- CD4+ T cells. Memory lymphocytes are formed after encountering a specific pathogen, and are vital for generating rapid and effective immune responses upon future encounters with the same pathogen [18]. We chose CD27 as a marker of memory status. CD27 expression is associated with activation in circulating B cells [9]. CD27+ B cells are larger and exhibit greater and more rapid proliferation and immunoglobulin production in response to antigenic stimulation [9]. In CD4+ T cells, loss of CD27 expression is seen in memory cells at a late stage in differentiation, and is associated with an increased capacity for IL-4 production [19]. Functional studies of CD4+ T cells report that CD27 expression distinguishes two distinct subpopulations, of which the CD27- subset shows a stronger antigen-recall response and increased cytokine secretion [10]. Relatively higher PD-1 expression on CD27- T cells may therefore have a greater negative effect on antigen-specific responses in both B cells and T cells, as there is T cell-dependent B cell development within the germinal centers of secondary lymphoid organs [20].

We measured soluble PD-1 (sPD-1) and sPD-L1 levels, as high levels of sPD-1 or sPD-L1 in sepsis could reduce the efficacy of anti-PD-1 or anti-PD-L1 antibody therapy by neutralisation. Furthermore, should serum levels correlate with cell surface expression this may offer a potential point-of-care biomarker to identify patients who could benefit from early PD-1 pathway blockade. We did not find any significant differences in sPD-1 or sPD-L1 levels between patients with sepsis and controls; of note, levels were towards the lower limit of detection in the majority of subjects. Previous studies measuring serum sPD-1/L levels in sepsis have yielded inconsistent results [21–23] (Additional file 17: Table S6). Importantly, concurrent cell surface expression was not measured in any of these studies. Timing of measurement may contribute to the differences; we measured sPD-1/PD-L1 within 12 h of ICU admission whereas the others varied from time of presentation to the emergency department [21] to within 24 h of ICU admission [22, 23]. Our pilot study results suggest that sPD-1 or sPD-L1 levels within 12 h of ICU admission do not identify patients with high cell surface PD-1/L expression [11]. This needs confirmation in larger cohorts, ideally using the same inclusion criteria as those planned for interventional trials.

In contrast to one previous study [7], we did not observe significant differences in PD-1/PD-L1 expression by either survival or nosocomial infection status. Aside from our small sample size, there are several alternative explanations. The kinetics of these checkpoint inhibitors is unknown in critically ill patients with sepsis. There is also a variable degree of immunosuppression even at the time of ICU admission though we specifically excluded any patients with previously documented immunosuppression. However, our timing of sampling within 12 h of ICU admission may be too early for differentiating survival status. This inference is supported by a recent study examining PD-1 expression by CD4+ T cells in a sepsis cohort using serial measurements on days 1, 3 and 7 of ICU admission, which found that while all septic patients had raised PD-1 at days 1 and 3, only survivors had normalised PD-1 expression by day 7 [17]. This highlights the need for further work to characterize how PD-1/L expression changes over the course of sepsis, how this relates to outcome, and the optimal recruitment window for any future trial of anti-PD-1 therapy. With regard to nosocomial infection, we found that lymphocyte PD-L1 expression was significantly higher in those who subsequently developed an infection, but only when the ICU length of stay exceeded 7 days. This may be explained by the competing risk of nosocomial infection with early ICU discharge or death. The additional risk provided by over-expression of these checkpoint inhibitors may be overwhelmed by stronger risk factors for mortality such as age, comorbidity and illness severity [14].

When interpreting our results, key limitations to consider include the small sample size, the use of healthy controls instead of non-sepsis critical illness controls, and that this was a post-hoc sub-study designed to test a hypothesis to inform future trials. We chose healthy controls as critically ill patients exhibit a range of immune deficits similar to those seen in patients with sepsis [24]; the use of non-infected critically ill controls could confound the association between PD-1 expression and outcomes. The key strengths of the study include the hypothesis-driven set of experiments that highlight the need for further research to define PD-1, PD-L1 and PD-L2 expression in sepsis, and its relationship to two competing events - nosocomial infection and death [11].

## Conclusions

In conclusion, our pilot study contributes to the further understanding of sepsis immunology by highlighting increased expression of these checkpoint regulators in B cells, and their differential expression by memory subset status in both B and T cells. The utility of CD27 status in lymphocytes as a putative biomarker for patient enrichment in anti PD-1 or anti PD-L1 trials warrants further study.

## Additional files

Additional file 1: **Figure S1.** Example of gating used in data analysis. The isotype (and FMO) was used to set the negative gate, and then the percentage of positive cells was taken as the percentage above this gate. Separate healthy and sepsis isotypes were used (Figure S1a and b respectively). Figure S1c shows an example of MFI signalling in healthy and sepsis isotypes, and healthy, sepsis survivor and sepsis non-survivor samples. (DOCX 441 kb)

Additional file 2: **Table S1.** Characteristics of patients with sepsis included in the study. Results are shown for all patients and for survivors and non-survivors (DOCX 14 kb)

Additional file 3: **Table S2.** Survival status, microbiology results and nosocomial infection site in patients who developed nosocomial infection. (DOCX 12 kb)

Additional file 4: **Table S3.** Proportion of positive B and T cells. Proportion of B and CD4+ T cells that express PD-1, PD-L1 and PD-L2 in patients with sepsis compared to healthy controls. (DOCX 12 kb)

Additional file 5: **Table S4.** Proportion of positive cells by CD27 expression status. Proportion of CD27+ B cells, CD27- B cells, CD27+ CD4+ T cells and CD27- CD4+ T cells that express PD-1, PD-L1 and PD-L2 in patients with sepsis compared to healthy controls. (DOCX 12 kb)

Additional file 6: **Figure S2.** B cell subset MFI. Comparison of expression of PD-1, PD-L1 and PD-L2 as determined by MFI on B cell subsets (CD27+ and CD27-) in patients with sepsis and healthy controls. (DOCX 251 kb)

Additional file 7: **Figure S3.** B cell percentage positivity. Comparison of the expression levels as determined by percentage positivity of PD-1, PD-L1 and PD-L2 on B cell subsets (CD27+ and CD27-) in patients with sepsis and healthy controls. (DOCX 468 kb)

Additional file 8: **Table S5.** MFI of PD-1, PD-L1 and PD-L2 on CD27+ B cells, CD27- B cells, CD27+ CD4+ T cells and CD27- CD4+ T cells, compared between patients with sepsis and healthy controls. (DOCX 12 kb)

Additional file 9: **Figure S4.** CD4+ T cell subset MFI. Comparison of expression of PD-1, PD-L1 and PD-L2 as determined by MFI on CD4+ T cell subsets (CD27+ and CD27-) between patients with sepsis and healthy controls. (DOCX 257 kb)

Additional file 10: **Figure S5.** CD4+ T cell percentage positivity. Comparison of the expression levels as determined by percentage positivity of PD-1, PD-L1 and PD-L2 on CD4+ T cell subsets (CD27+ and CD27-) between patients with sepsis and healthy controls. (DOCX 465 kb)

Additional file 11: **Figure S6.** Comparison by nosocomial infection status. Comparison of expression of PD-1, PD-L1 and PD-L2 between patients who developed a nosocomial infection and those who did not. (DOCX 253 kb)

Additional file 12: **Figure S7.** PD-L1 comparison by nosocomial infection status in patients with ICU length of stay ≥7 days. PD-L1 expression by lymphocytes was compared between patients who developed a nosocomial infection and those who did not, when patients with an ICU length of stay <7 days were excluded. (DOCX 24 kb)

Additional file 13: **Figure S8.** Comparison by nosocomial infection status in patients with ICU length of stay ≥7 days. Comparison of PD-1, PD-L1 and PD-L2 expression by B and CD4+ T cells between patients who developed a nosocomial infection and those who did not, when patients with an ICU length of stay <7 days were excluded. (DOCX 237 kb)

Additional file 14: **Figure S9.** Comparison by survival status. Comparison of PD-1, PD-L1 and PD-L2 expression by B and CD4+ T cells between sepsis survivors and non-survivors. (DOCX 278 kb)

Additional file 15: **Figure S10.** Serum level comparison. Comparison of levels of serum PD-1 and PD-L1 between patients with sepsis and healthy controls. (DOCX 175 kb)

Additional file 16: **Figure S11.** Serum versus cell surface expression. Serum levels of PD-1 and PD-L1 are plotted against cell surface expression levels on B cells and CD4+ T cells. (DOCX 286 kb)

Additional file 17: **Table S6.** sPD-1/sPD-L1 studies in sepsis. Summary of previous studies measuring soluble serum PD-1 and PD-L1 (sPD-1; sPD-L1) levels in sepsis. (DOCX 12 kb)

## Abbreviations

ELISA: Enzyme-linked immunosorbent assay
FCS: Flow cytometry standard
FMO: Fluorescence minus one
ICU: Intensive care unit
MFI: Mean fluorescence intensity
PD-1: Programmed death protein 1
PD-L1: Programmed death-ligand 1
PD-L2: Programmed death-ligand 2
SD: Standard deviation
sPD-1: Soluble programmed death protein 1
sPD-L1: Soluble programmed death-ligand 1
