# Supplementary material for: Lymphocyte subset expression and serum concentrations of PD-1/PD-L1 in sepsis - pilot study
Source: Crit Care. 2018 Apr 17;22:95. doi: 10.1186/s13054-018-2020-2 (PMC5902875; doi:10.1186/s13054-018-2020-2)
Supplement: Supplementary file 1 — Figure S1. Example of gating used in data analysis. The isotype (and FMO) was used to set the negative gate, and then the percentage of positive cells was taken as the percentage above this gate. Separate healthy and sepsis isotypes were used (Figure S1a and b respectively). Figure S1c shows an example of MFI signalling in healthy and sepsis isotypes, and healthy, sepsis survivor and sepsis non-survivor samples. (DOCX 441 kb) [file 13054_2018_2020_MOESM1_ESM.docx]

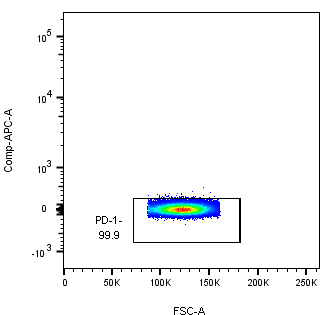

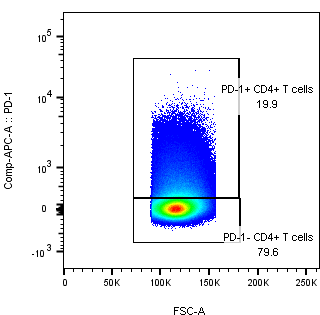


Healthy isotype

Healthy sample

**Figure S1a. Example of gating used in data analysis.**

Sepsis isotype

Sepsis sample


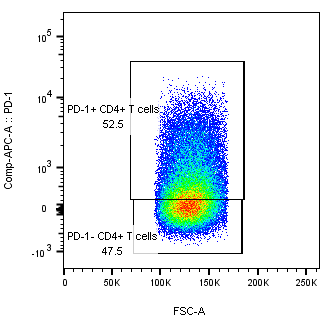

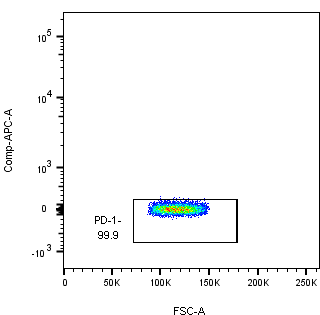


**eFigure 1b. Example of gating used in data analysis.**

**eFigure 1c. Example of gating used in data analysis.**
