# Supplementary material for: Lymphocyte subset expression and serum concentrations of PD-1/PD-L1 in sepsis - pilot study
Source: Crit Care. 2018 Apr 17;22:95. doi: 10.1186/s13054-018-2020-2 (PMC5902875; doi:10.1186/s13054-018-2020-2)
Supplement: Supplementary file 2 — Table S1. Characteristics of patients with sepsis included in the study. Results are shown for all patients and for survivors and non-survivors (DOCX 14 kb) [file 13054_2018_2020_MOESM2_ESM.docx]

|  | All (n=22) | Survivor (n=11) | Non-survivors (n=11) |
| --- | --- | --- | --- |
| Gender, number of males (percentage) | 16 (73%) | 10 (91%) | 6 (55%) |
| Age (years), median (IQR) | 68.5 (54.8 – 84.3) | 59 (48 – 71) | 81 (64 – 85) |
| Co-morbidities, number (percentage)   - Hypertension - Ischaemic heart disease - Atrial fibrillation - Cerebrovascular disease - Type 2 diabetes mellitus - Chronic kidney disease - Chronic obstructive pulmonary disease - Depression | 7 (63.6%)  3 (27.3%)  3 (27.3%)  3 (27.3%)  6 (54.5%)  2 (18.2%)  4 (36.4%)  3 (27.3%) | 2 (18.2%)  0  0  1 (9.1%)  3 (27.3%)  1 (9.1%)  2 (18.2%)  2 (18.2%) | 5 (45.5%)  3 (27.3%)  3 (27.3%)  2 (18.2%)  3 (27.3%)  1 (9.1%)  2 (18.2%)  1 (9.1%) |
| Source of sepsis, number (percentage)   - Respiratory - Intra-abdominal - Wound/soft tissue | 16 (73%)  4 (18%)  2 (9%) | 10 (91%)  1 (9%)  0 | 6 (55%)  3 (27%)  2 (18%) |
| SOFA score on admission, median (IQR) | 7 (5.0 – 8.3) | 7 (5.0 – 9.0) | 7 (6.0 – 8.0) |
| APACHE II score on admission, mean (SD) | 20.7 (5.8) | 17.8 (3.7) | 23.6 (6.1) |
| White cell count on admission, mean (SD) | 15.9 (7.8) | 15.5 (6.4) | 16.3 (9.3) |
| Lymphocyte count on admission, mean (SD) | 1.1 (0.7) | 1.2 (0.8) | 1.0 (0.6) |
| Neutrophil/lymphocyte ratio, mean (SD) | 18.8 (17.9) | 14.3 (8.2) | 23.3 (23.7) |
| ICU length of stay (days), median (IQR) | 10.5 (7.0 – 20.3) | 7.0 (3.0 – 11.0) | 14.0 (10.0 – 22.0) |

**Table S1. Sepsis patient characteristics.** Table showing characteristics of sepsis patients included in the study. Figures are shown for all patients, and for survivors and non-survivors.
