# Supplementary material for: Lymphocyte subset expression and serum concentrations of PD-1/PD-L1 in sepsis - pilot study
Source: Crit Care. 2018 Apr 17;22:95. doi: 10.1186/s13054-018-2020-2 (PMC5902875; doi:10.1186/s13054-018-2020-2)
Supplement: Supplementary file 3 — Table S2. Survival status, microbiology results and nosocomial infection site in patients who developed nosocomial infection. (DOCX 12 kb) [file 13054_2018_2020_MOESM3_ESM.docx]

| **Survival status** | **Site of nosocomial infection** | **Microbiology** |
| --- | --- | --- |
| Survivor | Chest | Staphylococcus aureus in sputum |
| Survivor | Abdomen | Klebsiella pneumoniae in blood cultures |
| Survivor | Chest | Candida albicans in sputum |
| Non-survivor | Urine | Klebsiella pneumoniae in urine |
| Non-survivor | Wound | Pseudomonas aeruginosa in wound swab |
| Non-survivor | Chest | Pseudomonas aeruginosa in sputum |
| Non-survivor | Abdomen | Candida albicans in urine  Clostridium difficile toxin positive |
| Non-survivor | No obvious source | Candida albicans in sputum |
| Non-survivor | Chest | Nil |
| Non-survivor | Abdomen | Nil |
| Non-survivor | Chest | Nil |

**Table S2.** **Infection site/microbiology of patients with nosocomial infections.** Table showing the survival status, microbiology results and nosocomial infection site of those patients who developed nosocomial infection.
