# Supplementary material for: Lymphocyte subset expression and serum concentrations of PD-1/PD-L1 in sepsis - pilot study
Source: Crit Care. 2018 Apr 17;22:95. doi: 10.1186/s13054-018-2020-2 (PMC5902875; doi:10.1186/s13054-018-2020-2)
Supplement: Supplementary file 4 — Table S3. Proportion of positive B and T cells. Proportion of B and CD4+ T cells that express PD-1, PD-L1 and PD-L2 in patients with sepsis compared to healthy controls. (DOCX 12 kb) [file 13054_2018_2020_MOESM4_ESM.docx]

|  | **B cells** | | | **CD4+ T cells** | | |
| --- | --- | --- | --- | --- | --- | --- |
|  | PD-1 | PD-L1 | PD-L2 | PD-1 | PD-L1 | PD-L2 |
| **Healthy** | 8.79 | 1.21 | 0.15 | 21.25 | 0.20 | 0.52 |
| **Sepsis** | 26.45 | 2.42 | 1.34 | 38.90 | 1.90 | 2.17 |
| **p-value** | 0.0002* | 0.0244* | 0.05 | 0.0023* | 0.0083* | 0.15 |

**Table S3.** Proportion of positive B and T cells. Table showing the proportion of B and CD4+ T cells which express PD-1, PD-L1 and PD-L2, compared between sepsis patients and healthy controls.
