# Supplementary material for: Lymphocyte subset expression and serum concentrations of PD-1/PD-L1 in sepsis - pilot study
Source: Crit Care. 2018 Apr 17;22:95. doi: 10.1186/s13054-018-2020-2 (PMC5902875; doi:10.1186/s13054-018-2020-2)
Supplement: Supplementary file 5 — Table S4. Proportion of positive cells by CD27 expression status. Proportion of CD27+ B cells, CD27- B cells, CD27+ CD4+ T cells and CD27- CD4+ T cells that express PD-1, PD-L1 and PD-L2 in patients with sepsis compared to healthy controls. (DOCX 12 kb) [file 13054_2018_2020_MOESM5_ESM.docx]

|  | **B cells** | | | | | |
| --- | --- | --- | --- | --- | --- | --- |
|  | CD27+ | | | CD27- | | |
|  | PD-1 | PD-L1 | PD-L2 | PD-1 | PD-L1 | PD-L2 |
| **Healthy** | 13.50 | 1.26 | 0.41 | 7.49 | 0.87 | 0.09 |
| **Sepsis** | 34.05 | 3.91 | 3.59 | 24.80 | 2.66 | 0.86 |
| **p-value** | **<0.0001*** | **0.0244*** | **0.0317*** | **0.0021*** | **0.0172*** | 0.34 |
|  | | | | | | |
|  | **CD4+ T cells** | | | | | |
|  | CD27+ | | | CD27- | | |
|  | PD-1 | PD-L1 | PD-L2 | PD-1 | PD-L1 | PD-L2 |
| **Healthy** | 19.80 | 0.09 | 0.65 | 49.20 | 0.21 | 0.96 |
| **Sepsis** | 35.15 | 1.90 | 2.71 | 70.45 | 2.08 | 2.02 |
| **p-value** | **0.0006*** | **0.0038*** | 0.16 | **0.0029*** | **0.0343*** | 0.47 |

**Table S4. Proportion of positive cells by CD27 expression status.** Table showing the proportion of CD27+ B cells, CD27- B cells, CD27+ CD4+ T cells and CD27- CD4+ T cells which express PD-1, PD-L1 and PD-L2, compared between sepsis patients and healthy controls.
