# Supplementary material for: Lymphocyte subset expression and serum concentrations of PD-1/PD-L1 in sepsis - pilot study
Source: Crit Care. 2018 Apr 17;22:95. doi: 10.1186/s13054-018-2020-2 (PMC5902875; doi:10.1186/s13054-018-2020-2)
Supplement: Supplementary file 6 — Figure S2. B cell subset MFI. Comparison of expression of PD-1, PD-L1 and PD-L2 as determined by MFI on B cell subsets (CD27+ and CD27-) in patients with sepsis and healthy controls. (DOCX 251 kb) [file 13054_2018_2020_MOESM6_ESM.docx]

**Figure S2. B cell subset MFI.** Box and whisker plots comparing expression by MFI of PD-1, PD-L1 and PD-L2 between sepsis patients and healthy controls on B cell subsets (CD27+ and CD27-).
