# Supplementary material for: Lymphocyte subset expression and serum concentrations of PD-1/PD-L1 in sepsis - pilot study
Source: Crit Care. 2018 Apr 17;22:95. doi: 10.1186/s13054-018-2020-2 (PMC5902875; doi:10.1186/s13054-018-2020-2)
Supplement: Supplementary file 8 — Table S5. MFI of PD-1, PD-L1 and PD-L2 on CD27+ B cells, CD27- B cells, CD27+ CD4+ T cells and CD27- CD4+ T cells, compared between patients with sepsis and healthy controls. (DOCX 12 kb) [file 13054_2018_2020_MOESM8_ESM.docx]

|  | **B cells** | | | | | |
| --- | --- | --- | --- | --- | --- | --- |
|  | CD27+ | | | CD27- | | |
|  | PD-1 | PD-L1 | PD-L2 | PD-1 | PD-L1 | PD-L2 |
| **Healthy** | 371 | 1566 | 1021 | 335 | 1622 | 972 |
| **Sepsis** | 575 | 1491 | 1189 | 472 | 1513 | 744 |
| **p-value** | **0.0001*** | 0.67 | 0.29 | **<0.0001*** | 0.25 | 0.36 |
|  | | | | | | |
|  | **CD4+ T cells** | | | | | |
|  | CD27+ | | | CD27- | | |
|  | PD-1 | PD-L1 | PD-L2 | PD-1 | PD-L1 | PD-L2 |
| **Healthy** | 625 | 989 | 629 | 685 | 1065 | 598 |
| **Sepsis** | 716 | 1327 | 925 | 972 | 1271 | 737 |
| **p-value** | **0.0163*** | **0.0190*** | 0.11 | **<0.0001*** | **0.0273*** | 0.39 |

**Table S5. MFI results.** Table showing the MFI of PD-1, PD-L1 and PD-L2 on CD27+ B cells, CD27- B cells, CD27+ CD4+ T cells and CD27- CD4+ T cells, compared between sepsis patients and healthy controls.
