# Supplementary material for: Lymphocyte subset expression and serum concentrations of PD-1/PD-L1 in sepsis - pilot study
Source: Crit Care. 2018 Apr 17;22:95. doi: 10.1186/s13054-018-2020-2 (PMC5902875; doi:10.1186/s13054-018-2020-2)
Supplement: Supplementary file 10 — Figure S5. CD4+ T cell percentage positivity. Comparison of the expression levels as determined by percentage positivity of PD-1, PD-L1 and PD-L2 on CD4+ T cell subsets (CD27+ and CD27-) between patients with sepsis and healthy controls. (DOCX 465 kb) [file 13054_2018_2020_MOESM10_ESM.docx]

**Figure S5. CD4+ T cell percentage positivity.** Dot plots comparing the expression levels by percentage positivity of PD-1, PD-L1 and PD-L2 between sepsis patients and healthy controls on CD4+ T cell subsets (CD27+ and CD27-).
