# Supplementary material for: Lymphocyte subset expression and serum concentrations of PD-1/PD-L1 in sepsis - pilot study
Source: Crit Care. 2018 Apr 17;22:95. doi: 10.1186/s13054-018-2020-2 (PMC5902875; doi:10.1186/s13054-018-2020-2)
Supplement: Supplementary file 11 — Figure S6. Comparison by nosocomial infection status. Comparison of expression of PD-1, PD-L1 and PD-L2 between patients who developed a nosocomial infection and those who did not. (DOCX 253 kb) [file 13054_2018_2020_MOESM11_ESM.docx]

**Figure S6.** **Comparison by nosocomial infection status.** Box and whisker plots comparing expression of PD-1, PD-L1 and PD-L2 between patients who developed a nosocomial infection and those who did not. *NI = nosocomial infection*
