# Supplementary material for: Lymphocyte subset expression and serum concentrations of PD-1/PD-L1 in sepsis - pilot study
Source: Crit Care. 2018 Apr 17;22:95. doi: 10.1186/s13054-018-2020-2 (PMC5902875; doi:10.1186/s13054-018-2020-2)
Supplement: Supplementary file 12 — Figure S7. PD-L1 comparison by nosocomial infection status in patients with ICU length of stay ≥7 days. PD-L1 expression by lymphocytes was compared between patients who developed a nosocomial infection and those who did not, when patients with an ICU length of stay <7 days were excluded. (DOCX 24 kb) [file 13054_2018_2020_MOESM12_ESM.docx]

**Figure S7. PD-L1 comparison by nosocomial infection status in patients with ICU length of stay ≥7 days.** Box and whisker plot showing PD-L1 expression by lymphocytes compared between patients who developed a nosocomial infection and those who did not, when patients with an ICU length of stay less than 7 days are excluded. *NI = nosocomial infection*
