# Supplementary material for: Lymphocyte subset expression and serum concentrations of PD-1/PD-L1 in sepsis - pilot study
Source: Crit Care. 2018 Apr 17;22:95. doi: 10.1186/s13054-018-2020-2 (PMC5902875; doi:10.1186/s13054-018-2020-2)
Supplement: Supplementary file 14 — Figure S9. Comparison by survival status. Comparison of PD-1, PD-L1 and PD-L2 expression by B and CD4+ T cells between sepsis survivors and non-survivors. (DOCX 278 kb) [file 13054_2018_2020_MOESM14_ESM.docx]

**Figure S9. Comparison by survival status.** Box and whisker plots comparing PD-1, PD-L1 and PD-L2 expression by B and CD4+ T cells between sepsis survivors and non-survivors.
