# Supplementary material for: Lymphocyte subset expression and serum concentrations of PD-1/PD-L1 in sepsis - pilot study
Source: Crit Care. 2018 Apr 17;22:95. doi: 10.1186/s13054-018-2020-2 (PMC5902875; doi:10.1186/s13054-018-2020-2)
Supplement: Supplementary file 15 — Figure S10. Serum level comparison. Comparison of levels of serum PD-1 and PD-L1 between patients with sepsis and healthy controls. (DOCX 175 kb) [file 13054_2018_2020_MOESM15_ESM.docx]

**Figure S10. Serum level comparison.** Dot plots comparing levels of serum PD-1 and PD-L1 between sepsis patients and healthy controls.
