# Supplementary material for: Lymphocyte subset expression and serum concentrations of PD-1/PD-L1 in sepsis - pilot study
Source: Crit Care. 2018 Apr 17;22:95. doi: 10.1186/s13054-018-2020-2 (PMC5902875; doi:10.1186/s13054-018-2020-2)
Supplement: Supplementary file 16 — Figure S11. Serum versus cell surface expression. Serum levels of PD-1 and PD-L1 are plotted against cell surface expression levels on B cells and CD4+ T cells. (DOCX 286 kb) [file 13054_2018_2020_MOESM16_ESM.docx]

**Figure S11. Serum vs cell surface expression.** Scatter graphs plotting serum levels of PD-1 and PD-L1 against cell surface expression levels on B cells and CD4+ T cells.
