# Supplementary material for: Lymphocyte subset expression and serum concentrations of PD-1/PD-L1 in sepsis - pilot study
Source: Crit Care. 2018 Apr 17;22:95. doi: 10.1186/s13054-018-2020-2 (PMC5902875; doi:10.1186/s13054-018-2020-2)
Supplement: Supplementary file 17 — Table S6. sPD-1/sPD-L1 studies in sepsis. Summary of previous studies measuring soluble serum PD-1 and PD-L1 (sPD-1; sPD-L1) levels in sepsis. (DOCX 12 kb) [file 13054_2018_2020_MOESM17_ESM.docx]

| **Study details** | **Patients** | **Measurement** | **Control group** | **Key findings** |
| --- | --- | --- | --- | --- |
| Zhao et al, 2017 [18] | Patients in ED with SIRS, sepsis, severe sepsis or septic shock (n=595) | sPD-1 measured at time of arrival in ED | Healthy controls (n=60) | sPD-1 higher in sepsis, severe sepsis and septic shock compared with controls |
| Liu et al, 2017 [19] | Patients with sepsis admitted to ICU (n=91) | sPD-1 and sPD-L1 measured on day 1, day 3-4 and day 7-8 of ICU admission | Healthy controls (n=29) | sPD-1 and sPD-L1 higher in sepsis compared to controls; sPD-L1 higher in non-survivors compared with survivors |
| Lange et al, 2017 [20] | Patients with sepsis admitted to ICU (n=101) | sPD-1 measured within 24 hrs of admission to ICU | Healthy controls (n=31); non-infected ICU patients (n=28) | sPD-1 lower in sepsis cohort compared to healthy controls; no significant difference compared to non-infected ICU patients |

**Table S6.** **sPD-1/sPD-L1 studies in sepsis.** Summary of previous studies measuring soluble serum PD-1 and PD-L1 (sPD-1; sPD-L1) levels in sepsis
